# Editorial

**DOI:** 10.1111/1751-7915.12058

**Published:** 2013-04-15

## Editors

**Kenneth N. Timmis** (Editor-in-Chief), Environmental Microbiology Laboratory, Helmholtz Center for Infection Research, Inhoffenstrasse 7, D-38124

Braunschweig, Germany. Tel.: +49 531 6181 4000; Fax: +49 531 6181 4199;

e-mail: kti@helmholtz-hzi.de

**Juan L. Ramos**, Estación Experimental del Zaidín – CSIC, C/ Prof. Albareda, 1, E-18008 Granada, Spain.

Tel.: +34 958 181 608; Fax: +34 958 135 740;

e-mail: jlramos@eez.csic.es

**Willem M. de Vos**, Laboratory of Microbiology, Wageningen University, Dreijenplein 10

Building 316, 6703 HB Wageningen, The Netherlands.

Tel.: +31 317 483 100; Fax: +31 317 483 829;

e-mail: willem.deVos@wur.nl

**Willy Verstraete**, Laboratory of Microbial Ecology and Technology (LabMET), Ghent University, Coupure Links 653, B-9000 Ghent, Belgium. Tel.: +32 9264 5976;

Fax: +32 9264 6248;

e-mail: willy.verstraete@ugent.be

**Martin Rosenberg**, Research & Development, Promega Corporation, 2800 Woods Hollow Road, Madison, WI 53711-5399, USA.

e-mail: martin.rosenberg@promega.com

REVIEWS AND SPECIAL ISSUE EDITOR

**Juan L. Ramos**, Estación Experimental

del Zaidín, Granada, Spain

WEB ALERTS

**Larry Wackett**, University of Minnesota, St Paul, USA

## Editorial Board

Lars Angenent, USA

Steven A. Banwart, UK

Shimshon Belkin, Israel

Giovanni Bertoni, Italy

Ian Booth, UK

Uwe Bornscheuer, Germany

Michael Bott, Germany

Harald Brussow, Switzerland

Rita Colwell, USA

Milton Costa, Portugal

Jiri Damborsky, Czech Replublic

Craig Daniels, Canada

Eduardo Diaz, Spain

Gerrit Eggink, Germany

Thomas Egli, Switzerland

Romilio Espejo, Chile

Manuel Ferrer, Spain

Julia Foght, Canada

Jim K Fredrickson, USA

Michael Galperin, USA

Jose Luis Garcia, Spain

Harry Gilbert, UK

Bernardo Gonzalez, Chile

Guido Grandi, Italy

Miguel G. Guerrero, Spain

Carlos A. Guzman, Germany

Ken Hammel, USA

Bernhard Hauer, Germany

Terry C. Hazen, USA

Michael Hecker, Germany

Hermann J. Heipieper, Germany

Monica Höfte, Belgium

Karl-Erich Jaeger, Germany

Dieter Jahn, Germany

Junichi Kato, Japan

Staffan Kjelleberg, Australia

Todd R. Klaenhammer, USA

Alexander M. Klibanov, USA

Rob Knight, USA

Tino Krell, Spain

Oscar Kuipers, The Netherlands

Robert Larossa, USA

Sang Yup Lee, Republic of Korea

Daniel van der Lelie, USA

Steven Lindow, USA

Mark van Loosdrecht, The Netherlands

Victor de Lorenzo, Spain

José M. Luengo, Spain

Ben Lugtenberg, The Netherlands

Mohamed A. Marahiel, Germany

Angel T. Martínez, Spain

Michael McInerney, USA

Rainer U. Meckenstock, Germany

Jan Roelof van der Meer, Switzerland

Diego de Mendoza, Argentina

Gabriella Molinari, Germany

Jens Nielsen, Sweden

Per Halkjær Nielsen, Denmark

Fergal O'Gara, Ireland

Sven Panke, Switzerland

David Payne, USA

Miguel A. Peñalva, Spain

Dietmar Pieper, Germany

Korneel Rabaey, Belgium

Lutgarde Raskin, USA

Fernando Rojo, Spain

Wilfred F.M. Röling, The Netherlands

Eliora Z. Ron, Israel

Sima Sariaslani, USA

Uwe Sauer, Switzerland

Bernhard Schink, Germany

Claudia Schmidt-Dannert, USA

Ana Segura, Spain

Roland J. Siezen, The Netherlands

Douwe van Sinderen, Ireland

Hauke Smidt, The Netherlands

## Editorial Board

Gloria Soberón Chávez, México

Alexander Steinbüchel, Germany

Bas Teusink, The Netherlands

Svein Valla, Norway

Erick Vandamme, Belgium

Jos Vanderleyden, Belgium

L. A. M. van der Wielen, The Netherlands

Rene H. Wijffels, The Netherlands

Roland Wohlgemuth, Switzerland

Thomas K. Wood, USA

Gary Woodnutt, USA

Michail Yakimov, Italy

Liping Zhao, China

## Scope

*Microbial Biotechnology* publishes papers of original research reporting significant advances in any aspect of microbial applications, including, but not limited to biotechnologies related to:

- microbial communities: structure:function relationships and communal behavior
- Green chemistry
- Primary metabolites
- Food, beverages and supplements
- Secondary metabolites and natural products
- Pharmaceuticals
- Diagnostics
- Agriculture
- Bioenergy
- Biomining, including oil recovery and processing
- Bioremediation
- Biopolymers, biomaterials
- Bionanotechnology
- Biosurfactants and bioemulsifiers
- Compatible solutes and bioprotectants
- Biosensors, monitoring systems, quantitative microbial risk assessment
- Technology development
- Protein engineering
- Functional genomics
- Metabolic engineering
- Metabolic design
- Systems analysis, modelling
- Process engineering
- Biologically-based analytical methods
- Microbially based strategies in public health
- Microbially based strategies to influence global processes

***Open Access and Copyright***. All articles published by *Microbial Biotechnology* are fully open access: immediately freely available to read, download and share. All *Microbial Biotechnology* articles are published under the terms of the Creative Commons Attribution License which permits use, distribution and reproduction in any medium, provided the original work is properly cited, and allows the commercial use of published articles.

Copyright on any research article in a journal published by *Microbial Biotechnology* is retained by the author(s). Authors grant Wiley a license to publish the article and identify itself as the original publisher. Authors also grant any third party the right to use the article freely as long as its integrity is maintained and its original authors, citation details and publisher are identified. Further information about open access license and copyright can be found on http://www.wileyopenaccess.com/details/content/12f25db4c87/Copyright--License.html.

***Use by commercial ‘for-profit’ organisations***. Use of Wiley Open Access articles for commercial, promotional, or marketing purposes requires further explicit permission fromWiley (corporatesales@wiley.com) and will be subject to a fee. Commercial purposes include:

- Copying or downloading of articles, or linking to such articles for further redistribution, sale or licensing;
- Copying, downloading or posting by a site or service that incorporates advertising with such content;

The inclusion or incorporation of article content in other works or services (other than normal quotations with an appropriate citation) that is then available for sale or licensing, for a fee (for example, a compilation produced for marketing purposes, inclusion in a salespack);

Use of article content (other than normal quotations with appropriate citation) by forprofit organisations for promotional purposes;

Linking to article content in e-mails redistributed for promotional, marketing or educational purposes;

Use for the purposes of monetary reward by means of sale, resale, licence, loan, transfer or other form of commercial exploitation such as marketing products;

Print reprints of Wiley Open Access articles can be purchased from corporatesales@wiley.com.

***Disclaimer***. The Publisher, Society for Applied Microbiology and Editors cannot be held responsible for errors or any consequences arising from the use of information contained in this journal; the views and opinions expressed do not necessarily reflect those of the Publisher, Society for Applied Microbiology and Editors; neither does the publication of advertisements constitute any endorsement by the Publisher, Society for Applied Microbiology and Editors of the products advertised.

Wiley Open Access articles posted to repositories or websites are without warranty from Wiley of any kind, either express or implied, including, but not limited to, warranties of merchantability, fitness for a particular purpose, or non-infringement. To the fullest extent permitted by law Wiley disclaims all liability for any loss or damage arising out of, or in connection with, the use of or inability to use the content.

